# Constructive Hybrid Games

**DOI:** 10.1007/978-3-030-51074-9_26

**Published:** 2020-05-30

**Authors:** Brandon Bohrer, André Platzer

**Affiliations:** 8 CNRS, LIG, Université Grenoble Alpes, Saint Martin d’Hères, France; 9 University Koblenz-Landau, Koblenz, Germany; 10 Computer Science Department, Carnegie Mellon University, Pittsburgh, USA; 11 Fakultät für Informatik, Technische Universität München, Munich, Germany

**Keywords:** Game logic, Constructive logic, Hybrid games, Dependent types

## Abstract

Hybrid games combine discrete, continuous, and adversarial dynamics. Differential game logic () enables proving (classical) existence of winning strategies. We introduce *constructive differential game logic* (CdGL) for hybrid games, where proofs that a player can win the game correspond to *computable* winning strategies. This constitutes the logical foundation for synthesis of correct control and monitoring code for safety-critical cyber-physical systems. Our contributions include novel semantics as well as soundness and consistency.

## Introduction

Differential Game Logic () provides a calculus for proving the (classical) existence of winning strategies for hybrid games [42], whose mixed discrete, continuous, and adversarial dynamics are compelling models for cyber-physical systems (CPSs). Classical existence does *not* necessarily imply that the resulting winning strategies are computable, however. To overcome this challenge, this paper introduces *Constructive Differential Game Logic* (CdGL) with a Curry-Howard correspondence: constructive proofs for constructive hybrid games correspond to programs implementing their winning strategies. We develop a new type-theoretic semantics which elucidates this correspondence and an operational semantics which describes the execution of strategies. Besides its theoretical appeal, this Curry-Howard interpretation provides the foundation for proof-driven synthesis methods, which excel at synthesizing expressive classes of games for which synthesis and correctness require interactive proof. Hybrid games are a compelling domain for proof-based synthesis both because many CPS applications are safety-critical or even life-critical, such as transportation systems, energy systems, and medical devices and because the combination of discrete, continuous, and adversarial dynamics makes verification and synthesis undecidable in both theory and practice. Our example model and proof, while short, lay the groundwork for future case studies.

*Challenges and Contributions.* In addition to  [42], we build directly on Constructive Game Logic (CGL) [9] for discrete games. Compared to CGL, we target a domain with readily-available practical applications (hybrid games), and introduce new type-theoretic and operational semantics which complement the realizability semantics of CGL while making Curry-Howard particularly clear and providing a simple notion of strategy execution. We overcome the following challenges in the process:

- Our semantics must carefully capture the meaning of constructive hybrid game strategies, including strategies for differential equations (ODEs).
- Soundness must be justified *constructively*. We adapt previous arguments to use constructive analysis [6, 12] by appealing to constructive formalizations of ODEs [17, 34]. This adaptation to our new semantics makes it possible to simplify statements of some standard lemmas.
- We study 1D driving control as an example, which demonstrates the strengths of both games and constructivity. Games and constructivity both introduce uncertainties: A player is uncertain how their opponent will play, while constructive real-number comparisons are never sure of exact equality. These uncertainties demand more nuanced proof invariants, but these nuances improve our fidelity to real systems.

These contributions are of likely interest to several communities. Other constructive program logics could reuse our semantic approach. Our example uses reach-avoid proofs for hybrid games, a powerful, under-explored [48] approach.

## Related Work

We discuss related works on games, constructive logic, and hybrid systems.

*Games in Logic.* Propositional GL was introduced by Parikh [39]. GL is a *program logic* in the spirit of Hoare calculi [26] or especially dynamic logics (DL) [47]: modalities capture the effect of game execution. GLs are unique in their clear delegation of strategy to the *proof* language rather than the *model* language, allowing succinct game specifications with sophisticated winning strategies. Succinct specifications are important: specifications are *trusted* because proving the *wrong theorem* would not ensure correctness. Relatives without this separation include SL [14], ATL [2], CATL [27], SDGL [23], structured strategies [49], DEL [3, 5, 56], evidence logic [4], and Angelic Hoare Logic [35].

*Constructive Modal Logics.* We are interested in the semantics of games, thus we review constructive modal semantics generally. This should not be confused with game semantics [1], which give a semantics to programs *in terms of* games. The main semantic approaches for constructive modal logics are intuitionistic Kripke semantics [58] and realizability semantics [32, 38]. CGL [9] used a realizability semantics which operate on a state, reminiscent of state in Kripke semantics, whereas we interpret CdGL formulas into type theory.

Modal Curry-Howard is relatively little-studied, and each author has their own emphasis. Explicit proof terms are considered for CGL [9] and a small fragment thereof [30]. Others [13, 18, 59] focus on intuitionistic semantics for their logics, fragments of CGL. Our semantics should be of interest for these fragments. We omit proof terms for space. CdGL proof terms would extend CGL proof terms [9] with a constructive version of existing classical ODE proof terms [8]. Propositional modal logic [37] has been interpreted as a type system.

*Hybrid Systems Synthesis.* Hybrid games synthesis is one motivation of this work. Synthesis of hybrid *systems* (1-player games) is an active area. The unique strength of proof-based synthesis is expressiveness: it can synthesize every provable system. CdGL proofs support first-order regular games with first-order (e.g., semi-algebraic) initial and goal regions. While synthesis and proof are both undecidable, interactive proof for undecidable logics is well-understood. The ModelPlex  [36] synthesizer for CdGL’s classical systems predecessor dL [44] recently added [11] proof-based synthesis to improve expressiveness. CdGL aims to provide a computational foundation for a more systematic proof-based synthesizer in the more general context of games.

Fully automatic synthesis, in contrast, restricts itself to small fragments in order to sidestep undecidability. Studied classes include rectangular hybrid games [25], switching systems [52], linear systems with polyhedral sets [31, 52], and discrete abstractions [20, 21]. A well-known [55] *systems* synthesis approach translates specifications *into* finite-alternation games. Arbitrary first-order games are our *source* rather than *target* language. Their approach is only known to terminate for simpler classes [50, 51].

## Constructive Hybrid Games

Hybrid games in CdGL are 2-player, zero-sum, and perfect-information, where continuous subgames are ordinary differential equations (ODEs) whose duration is chosen by a player. Hybrid games should not be confused with *differential games* which compete continuously [29, 43]. The players considered in this paper are Angel and Demon where the player currently controlling choices is always called Angel, while the player waiting to play is always called Demon. For any game $\alpha$ and formula $\phi ,$ the modal formula  says Angel can play $\alpha$ to ensure postcondition $\phi ,$ while  says Demon can play $\alpha$ to ensure postcondition $\phi$. These generalize safety and liveness modalities from DL. Dual games  unique to GLs, take turns by switching the Angel and Demon roles in game $\alpha$. The Curry-Howard interpretation of a proof of a CdGL modality  or  is a program which performs each player’s winning strategy. Games can have several winning strategies, each corresponding to a different proof and a different program.

### Syntax of CdGL

We introduce the language of CdGL with three classes of expressions *e*: terms *f*, *g*,  games $\alpha ,\beta ,$ and formulas $\phi , \psi .$ We characterize terms semantically for the sake of generality: a shallow embedding of CdGL inside a proof assistant might use the host language for terms. For games and formulas, we find it more convenient to explicitly and syntactically define a closed language.

A (scalar) semantic term is a function from states to reals, which are understood constructively *à la* Bishop [6, 12]. We use Bishop-style real analysis because it preserves many classical intuitions (e.g., uncountability) about $\mathbb {R}$ while ensuring computability. Type-2 [57] computability requires that all *functions on real numbers* are computable to arbitrary precision if represented as streams of bits, yet computability *does not* require that variables range over *only* computable reals. It is a theorem [57] that all such computable functions are continuous, but not always Lipschitz-continuous nor differentiable.

We introduce commonly used term constructs, which are not exhaustive because the language of terms is open. The simplest terms are *game variables* $x, y \in \mathcal {V}$ where $\mathcal {V}$ is the (at most countable) set of variable identifiers. The game variables, which are mutable, contain the state of the game, which is globally scoped. For every base game variable *x* there is a primed counterpart ${x'}$ whose purpose within an ODE is to track the time derivative of *x*. Real-valued terms *f*, *g* are simply type-2 computable functions, usually from states to reals. It is occasionally useful for *f* to return a tuple of reals, which are computable when every component is computable. Since terms are functions, operators are combinators: $f + g$ is a function which sums the results of *f* and *g*.

#### Definition 1

**(Terms).** A *term* *f*, *g* is any computable function over the game state. The following constructs appear in this paper:

$$\begin{aligned} f,g ~\mathrel {::=}~ \cdots ~|~c ~|~x ~|~f + g ~|~f \cdot g ~|~f / g ~|~\min (f,g) ~|~\max (f,g) ~|~(f)' \end{aligned}$$

where $c \in \mathbb {R}$ is a real literal, *x* a game variable, $f + g$ a sum, $f \cdot g$ a product, and $f / g$ is real division of *f* by *g*. Divisors *g* are assumed to be nonzero. Minimum and maximum of terms *f* and *g* are written $\min (f,g)$ and $\max (f,g)$. Any differentiable term *f* has a definable (Sect. 4.2) spatial differential term $(f)',$ which agrees with the time derivative within an ODE.

CdGL is constructive, so Angel strategies make choices computably. Until his turn, Demon just observes Angel’s choices, and does not care whether Angel made them computably. We discuss game-playing informally here, then formally in Sect. 4. In  are the ODE and dual games, which respectively distinguish hybrid games from discrete games and games from systems.

#### Definition 2

**(Games).** The set of *games* $\alpha ,\beta$ is defined recursively as such:


The *test game*  is a no-op if Angel proves $\phi ,$ else Demon wins by default since Angel “broke the rules”. A deterministic assignment  updates game variable *x* to the value of term *f*. Nondeterministic assignments  ask Angel to compute the new value of $x : \mathbb {R},$ i.e., Angel’s strategy for  is a term whose value is assigned to *x*. The ODE game  evolves ODE ${x'}=f$ for duration $d \ge 0$ chosen by Angel such that Angel proves the domain constraint formula $\psi$ is true throughout. We require that term *f* is effectively-locally-Lipschitz on domain $\psi$, meaning that at every state satisfying $\psi ,$ a neighborhood and coefficient *L* can be constructed such that *L* is a Lipschitz constant of *f* in the neighborhood. Effective local Lipschitz continuity guarantees unique solutions exist by constructive Picard-Lindelöf [34]. ODEs are explicit-form, so no primed variable ${y'}$ for $y \in \mathcal {V}$ is mentioned in *f* or $\psi$. Systems of ODEs are supported, we present single equations for readability. In the choice game $\alpha \cup \beta ,$ Angel chooses whether to play game $\alpha$ or game $\beta$. In the sequential composition game $\alpha ;\beta$, game $\alpha$ is played first, then $\beta$ from the resulting state. In the repetition game  Angel chooses after each repetition of $\alpha$ whether to continue playing, but must not repeat $\alpha$ infinitely. The exact number of repetitions is not known in advance, because it may depend on Demon’s reactions. In the dual game  Angel takes the Demon role and vice-versa while playing $\alpha$. Demon strategies “wait” until a dual game  is encountered, then play an Angelic strategy for $\alpha$. We parenthesize games with braces $\{ \alpha \}$ when necessary.

#### Definition 3

**(CdGL** **Formulas).** The CdGL *formulas* $\phi$ (also $\psi$) are:


Above, $f \sim g$ is a comparison formula for ${\sim }\mathrel {\in }\{\le , <, =, \ne , >, \ge \}$. The defining formulas of CdGL (and GL) are the modalities  and . These mean that Angel or Demon respectively have a *constructive* strategy to play hybrid game $\alpha$ and prove postcondition $\phi$. We do not develop modalities for existence of classical strategies because those cannot be synthesized to executable code.

Standard connectives are defined from games and comparisons. Verum ($\mathtt{tt}$) is defined $1 > 0$ and falsum ($\mathtt{ff}$) is $0 > 1$. Conjunction $\phi \wedge \psi$ is defined  disjunction $\phi \vee \psi$ is defined  and implication $\phi \rightarrow \psi$ is defined . Real quantifiers  and  are defined  and  respectively. As usual, equivalence $\phi \leftrightarrow \psi$ reduces to $(\phi \rightarrow \psi ) \wedge (\psi \rightarrow \phi ),$ negation $\lnot \phi$ is defined as $\phi \rightarrow \mathtt{ff}$, and inequality is defined by $f \ne g \equiv \lnot (f = g)$. Semantics and proof rules are needed only for core constructs, but we use derived constructs when they improve readability. Keep these definitions in mind, because the semantics and rules for some game connectives mirror first-order connectives.

For convenience, we also write derived operators where Demon is given control of a single choice before returning control to Angel. The *Demonic choice*  defined  says Demon chooses which branch to take, but Angel controls the subgames. *Demonic repetition*  is defined likewise by .

We write  (likewise for $\alpha$ and *f*) for the *renaming* of variable *x* for *y* and vice versa in formula $\phi$, and write  for the result of *substitution* of term *f* for game variable *x* in $\phi$, if the substitution is admissible (Definition 12 on page 14).

### Example Game

We give an example game and theorem statements, proven in [10]. Automotive systems are a major class of CPS. As a simple indicative example we consider time-triggered 1-dimensional driving with adversarial timing. For maximum time *T* between control cycles, we let Demon choose any duration in [0, *T*]. When we need to prohibit pathological “Zeno” behaviors while keeping constraints realistic, we can further restrict $t \in [T/2, T]$.

We write *x* for the current position of the car, *v* for its velocity, *a* for the acceleration, $A > 0$ for the maximum positive acceleration, and $B > 0$ for the maximum braking rate. We assume $x=v=0$ initially to simplify arithmetic. In time-triggered control, the controller runs at least once every $T > 0$ time units. Time and physics are continuous, *T* gives an upper bound on how often the controller runs. Local clock *t* marks the current time within the current timestep, then resets at each step. The control game ($\textsf {ctrl}$) says Angel can pick any acceleration *a* that is physically achievable ($-B \le a \le A$). The clock *t* is then reinitialized to 0. The plant game ($\textsf {plant}$) says Demon can evolve physics for duration $t \in [0,T]$ such that $v \ge 0$ throughout, then returns control to Angel.

Typical theorems in DLs and GLs are *safety* and *liveness*: are unsafe states always avoided and are desirable goals eventually reached? Safety and liveness of the 1D *system* has been proved previously: safe driving ($\textsf {safety}$) never goes past goal *g*,  while live driving eventually reaches *g* ($\textsf {liveness}$).Liveness theorem $\textsf {liveness}$ requires a lower time bound () to rule out Zeno strategies where Demon “cheats” by exponentially decreasing durations to essentially freeze the progress of time. The limit $t \ge T/2$ is chosen for simplicity. Safety theorem $\textsf {safety}$ omits this constraint because even Zeno behaviors are safe.

Safety and liveness theorems, if designed carelessly, have trivial solutions including but not limited to Zeno behaviors. It is safe to remain at $x=0$ and is live to maintain $a = A,$ but not vice-versa. In contrast to DLs, GLs easily express the requirement that *the same* strategy is both safe and live: we must remain safe *while* reaching the goal. We use this *reach-avoid* specification because it is immune to trivial solutions. We give a new reach-avoid result for 1D driving.

#### Example 4

*(Reach-avoid).* The following is provable in  and CdGL:


Angel *reaches* $g=x \wedge v=0$ while safely *avoiding* states where $x \le g$ does not hold. Angel is safe at *every* iteration for *every* time $t \in [0,T]$, thus safe *throughout* the game. The (dual) test  appears second, allowing Demon to win if Angel violates safety during $t < T/2$.

**Fig. 1.**
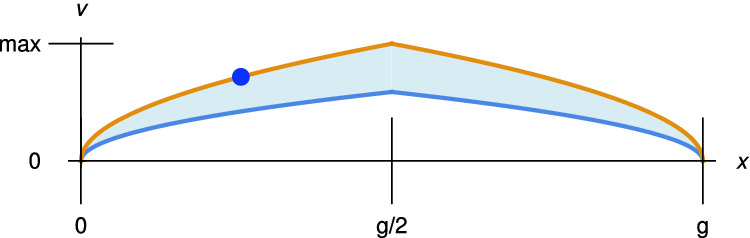
Safe driving envelope (Color figure online)

1D driving is well-studied for classical systems, but the constructive reach-avoid proof [10] is subtle. The proof constructs an envelope of safe upper and live lower bounds on velocity as a function of position (Fig. 1). The blue point indicates where Angel must begin to brake to ensure time-triggered safety. It is surprising that Angel can achieve postcondition $g=x \wedge v=0$, given that trichotomy ($f < g \vee f = g \vee f > g$) is constructively invalid. The key [10] is that comparison *terms* $\min (f,g)$ and $\max (f,g)$ *are* exact in Type 2 computability where bits of $\min$ and $\max$ may be computed lazily. Our exact result encourages us that constructivity is not overly burdensome in practice. When decidable comparisons ($f < g + \delta \vee f > g$) *are* needed, the alternative is a weaker guarantee $g-\varepsilon \le x \le g$ for parameter $\varepsilon > 0$. This relaxation is often enough to make the theorem provable, and reflects the fact that real agents only expect to reach their goal within finite precision.

## Type-Theoretic Semantics

In this section, we define the semantics of hybrid games and game formulas in type theory. We start with assumptions on the underlying type theory.

### Type Theory Assumptions

We assume a Calculus of Inductive and Coinductive Constructions (CIC)-like type theory [15, 16, 54] with polymorphism and dependency. We write *M* for terms and  to say *M* has type $\tau$ in CIC context $\varDelta$. We assume first-class (indexed [19]) inductive and coinductive types. We write $\tau$ for type families and $\kappa$ for kinds: type families inhabited by other type families. Inductive type families are written $\mu {t:\kappa }.\,{\tau },$ which denotes the *smallest* solution ty of kind $\kappa$ to the fixed-point equation  Coinductive type families are written $\rho {t:\kappa }.\,{\tau },$ which denotes the *largest* solution ty of kind $\kappa$ to the fixed-point equation  Type-expression $\tau$ must be monotone in *t* so smallest and largest solutions exist by Knaster-Tarski [24, Thm. 1.12]. Proof assistants like Coq reject definitions where monotonicity requires nontrivial proof; we did not mechanize our proofs because they use such definitions.

We use one predicative universe which we write $\mathbb {T}$ and Coq writes Type 0. Predicativity is an important assumption because our semantic definition is a large elimination, a feature known to interact dangerously with impredicativity. We write $\mathrm{{\Pi }} x\mathrel {:}\tau _1.\,\tau _2$ for a dependent function type with argument named *x* of type $\tau _1$ and where return type $\tau _2$ may mention *x*. We write $\mathrm{{\Sigma }} x\mathrel {:}\tau _1.\,\tau _2$ for a dependent pair type with left component named *x* of type $\tau _1$ and right component of type $\tau _2,$ possibly mentioning *x*. These specialize to the simple function $\tau _1 \Rightarrow \tau _2$ and product types $\tau _1\,\texttt {*}\,\tau _2$ respectively when *x* is not mentioned in $\tau _2$. Lambdas $(\lambda x:\tau .\, M)$ inhabit dependent function types. Pairs (*M*, *N*) inhabit dependent pair types. Application is $M\ N$. Let-binding unpacks pairs, whose left and right projection are $\pi _{L}M$ and $\pi _{R}M$. We write $\tau _1 + \tau _2$ for a disjoint union inhabited by $\ell \cdot M$ and $r \cdot M,$ and write $\textsf {case}\ A\ \textsf {of}~ p \Rightarrow B~|~ q \Rightarrow C$ for its case analysis.

We assume a real number type $\mathbb {R}$ and a Euclidean state type . The positive real numbers are written $\mathbb {R} _{>0}$, nonnegative reals $\mathbb {R} _{\ge 0}$. We assume scalar and vector sums, products, inverses, and units. States *s*, *t* support operations ${s}\ x$ and ${{\textsf {set}}}\ {s}\ x\ v$ which respectively retrieve the value of variable *x* in  or update it to *v*. The usual axioms of setters and getters [22] are satisfied. We write  for the distinguished variable of type  representing the current state. We will find it useful to consider the semantics of an expression both at current state  and at states *s*, *t* defined in terms of  (e.g., ).

### Semantics of CdGL

Terms *f*, *g* are type-theoretic functions of type . We will need differential terms $(f)',$ a definable term construct when *f* is differentiable. Not every term *f* need be differentiable, so we give a *virtual* definition, defining when $(f)'$ is equal to some term *g*. If $(f)'$ does not exist, then $(f)' = g$ is not provable. We define the (total) differential as the Euclidean dot product (${\varvec{\cdot }}$) of the gradient (variable name: $\nabla$) with ${s'},$ which is the vector of values $s\, {x'}$ assigned to primed variables ${x'}$. To show that $\nabla$ is the gradient, we define the gradient as a limit, which we express in $(\varepsilon ,\delta )$ style. In this definition, *f* and *g* are scalar-valued, and the minus symbol is used for both scalar and vector difference.For practical proofs, a library of standard rules for automatic, syntactic differentiation of common arithmetic operations [7] could be proven.

The interpretation  of formula $\phi$ is a predicate over states. A predicate of kind  is also understood as a *region*, e.g.,  is the region containing states where $\phi$ is provable. A CdGL context $\varGamma$ is interpreted over a uniform state term  where  i.e., *s* usually mentions . We define  to be the CIC context containing  and  for each $\phi \in \varGamma$. The sequent $(\varGamma \vdash \phi )$ is *valid* if there exists *M* where . Formula $\phi$ is *valid* iff sequent  is valid. That is, a valid formula is provable in every state with a common proof term *M*. The witness may inspect the state, but must do so constructively. Formula semantics employ the Angelic and Demonic semantics of games, which determine how to win a game $\alpha$ whose postcondition is $\phi$. We write  for the Angelic semantics of $\alpha$ and  for its Demonic semantics.

#### Definition 5

**(Formula semantics).** Angel and Demon strategies for a hybrid game $\alpha$ with goal region *P* are inhabitants of $\langle \!\langle {\alpha }\rangle \!\rangle \ P$ and $[[{\alpha }]]\ P,$ respectively.


Modality  is provable in *s* when  is inhabited so Angel has an $\alpha$ strategy from *s* to reach region  on which $\phi$ is provable. Modality  is provable in *s* when  is inhabited so Demon has an $\alpha$ strategy from *s* to reach region  on which $\phi$ is provable. For ${\sim } \mathrel {\in } \{\le ,<,=,\ne ,>,\ge \},$ the values of *f* and *g* are compared at state *s* in $f \sim g$. The game and formula semantics are simultaneously inductive. In each case, the connectives which define $\langle \!\langle {\alpha }\rangle \!\rangle$ and $[[{\alpha }]]$ are duals, because  and  are dual. Below, *P* refers to the goal region of the game and *s* to the initial state.

#### Definition 6

**(Angel semantics).** We define  inductively (by a large elimination) on $\alpha$:
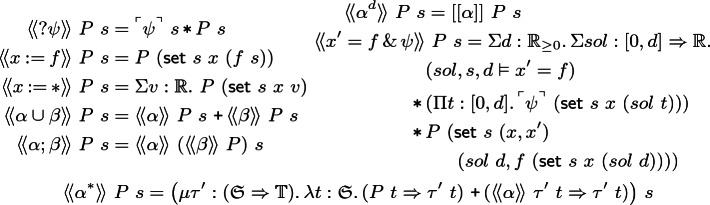


Angel wins  by proving both $\psi$ and *P* at *s*. Angel wins the deterministic assignment  by performing the assignment, then proving *P*. Angel wins nondeterministic assignment  by constructively choosing a value *v* to assign, then proving *P*. Angel wins $\alpha \cup \beta$ by choosing between playing $\alpha$ or $\beta ,$ then winning that game. Angel wins $\alpha ;\beta$ if she wins $\alpha$ with the postcondition of winning $\beta$. Angel wins  if she wins $\alpha$ in the Demon role. Angel wins ODE game  by choosing some solution *sol* of some duration $d$ which satisfies the ODE and domain constraint throughout and the postcondition $\phi$ at time $d$. While top-level postconditions rarely mention ${x'},$ intermediate invariant steps do, thus *x* and ${x'}$ both are updated in the postcondition. The construct $(sol,s,d \vDash {x'}=f),$ saying *sol* solves ${x'}=f$ from state *s* for time $d$, is defined:

$${(sol,s,d \vDash {x'}=f) \equiv \big ( ({s}\ x = sol\ 0)\,\texttt {*}\,\mathrm{{\Pi }} r\mathrel {:}[0,d].\, ((sol)'\ r = f\ ({{\textsf {set}}}\ {s}\ x\ (sol\ r))) \big )}$$

Note that variable *sol* stands for a function of the host theory, all of which are computable and therefore continuous. When $(sol,s,d \vDash {x'}=f)$ holds, *sol* is also continuously differentiable. Constructive Picard-Lindelöf [34] constructs a solution for every effectively-locally-Lipschitz ODEs, which need not have a closed form. The proof calculus we introduce in Sect. 5 includes both solution-based proof rules, which are useful for ODEs with simple closed forms, and invariant-based rules, which enable proof even when closed forms do not exist.

Angel strategies for  are inductively defined: either choose to stop the loop and prove *P* now, else play a round of $\alpha$ before repeating inductively. By Knaster-Tarski [24, Thm. 1.12], this least fixed point exists because the interpretation of a game is monotone in its postcondition (Lemma 7).

#### Lemma 7

**(Monotonicity).** Let . If  then there exists a term *N* such that 

#### Definition 8

**(Demon semantics).** We define  inductively (by a large elimination) on $\alpha$:
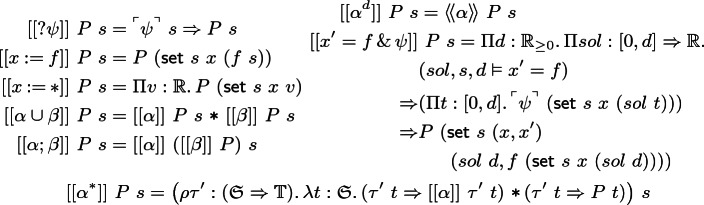


Demon wins  by proving *P* under assumption $\psi$, which Angel must provide (Sect. 7). Demon’s deterministic assignment is identical to Angel’s. Demon wins  by proving $\psi$ for *every* choice of *x*. Demon wins $\alpha \cup \beta$ with a pair of winning strategies. Demon wins $\alpha ;\beta$ by winning $\alpha$ with a postcondition of winning $\beta$. Demon wins  if he can win $\alpha$ after switching roles with Angel. Demon wins  if for an arbitrary duration and arbitrary solution which satisfy the domain constraint, he can prove the postcondition. Demon wins  if he can prove *P* no matter how many times Angel makes him play $\alpha$. Demon repetition strategies are coinductive using some invariant $\tau '$. When Angel decides to stop the loop, Demon responds by proving *P* from $\tau '$. Whenever Angel chooses to continue, Demon proves that $\tau '$ is preserved. Greatest fixed points exist by Knaster-Tarski [24, Thm. 1.12] using Lemma 7.

It is worth comparing the Angelic and Demonic semantics of . An Angel strategy says how to compute *x*. A Demon strategy simply accepts $x \in \mathbb {R}$ as its input, even uncomputable numbers. This is because Angel strategies supply a computable real while Demon acts with computable outputs given real *inputs*. In general, each strategy is constructive but permits its opponent to play classically. In the cyber-physical setting, the opponent is indeed rarely a computer.

## Proof Calculus

To enable direct syntactic proof, we give a natural deduction-style system for CdGL. We write $\varGamma = \psi _1, \ldots , \psi _n$ for a context of formulas and $\varGamma \vdash \phi$ for the natural-deduction sequent with conclusion $\phi$ and context $\varGamma$. We begin with rules shared by CGL [9] and CdGL, then give the ODE rules. We write  for the renaming of game variable *x* to *y* and vice versa in context $\varGamma$. Likewise  is the substitution of term *f* for game variable *x*. To avoid repetition, we write  to indicate that the same rule applies for  and . These rules write  for the dual of . We write $\mathop {\text {FV}}(e),$ $\mathop {\text {BV}}(\alpha ),$ and $\mathop {\text {MBV}}(\alpha )$ for the free variables of expression *e*,  bound variables of game $\alpha ,$ and must-bound variables of game $\alpha$ respectively, i.e., variables which *might* influence the meaning of an expression, might be modified during game execution, or are written during *every* execution.

**Fig. 2.**
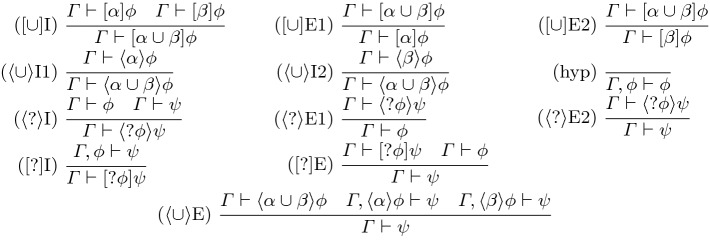
CdGL proof calculus: propositional game rules

Figure 2 gives the propositional game rules. Rule [?]E is modus ponens and [?]I is implication introduction because  is implication. Angelic choices are disjunctions introduced by $\langle \cup \rangle$I1 and $\langle \cup \rangle$I2 and case-analyzed by $\langle \cup \rangle$E. Angelic tests and Demonic choices are conjunctions introduced by $\langle ? \rangle$I and $[\cup ]$I, eliminated by $\langle ? \rangle$E1, $\langle ? \rangle$E2, $[\cup ]$E1, and $[\cup ]$E2. Rule hyp applies an assumption.

**Fig. 3.**
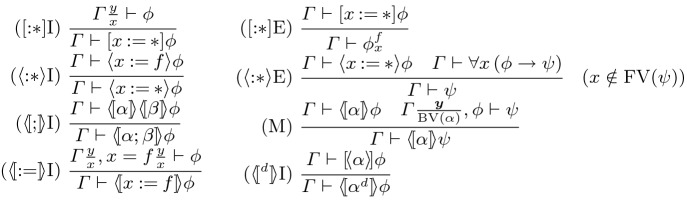
CdGL proof calculus: first-order games (*y* fresh, *f* computable,  admissible)

Figure 3 covers assignment, choice, sequencing, duals, and monotonicity. Angelic games have injectors ($\langle * \rangle \mathrm{S},\langle * \rangle \mathrm{G}$) and case analysis ($\langle * \rangle \mathrm{E}$). Duality  switches players by switching modalities. Sequential games  are decomposed as nested modalities.

Monotonicity (M) is Lemma 7 in rule form. The second premiss writes  to indicate that the bound variables of $\alpha$ must be freshly renamed in $\varGamma$ for soundness. Rule M is used for generalization because all GLs are subnormal, lacking axiom K (modal modus ponens) and necessitation. Common uses include concise right-to-left symbolic execution proofs and, in combination with  , Hoare-style sequential composition reasoning.

Nondeterministic assignments quantify over real-valued game variables. Assignments  remember the initial value of *x* in fresh variable *y* () for sake of completeness, then provide an assumption that *x* has been assigned to *f*. Skolemization $[:*]$I bound-renames *x* to *y* in $\varGamma$, written . Specialization $[:*]$E instantiates *x* to a term *f* by substituting . Existentials are introduced by giving a witness *f* in $\langle {:\!*}\rangle$I. Herbrandization $\langle {:\!*}\rangle$E unpacks existentials, soundness requires *x* is not free in $\psi$.

**Fig. 4.**
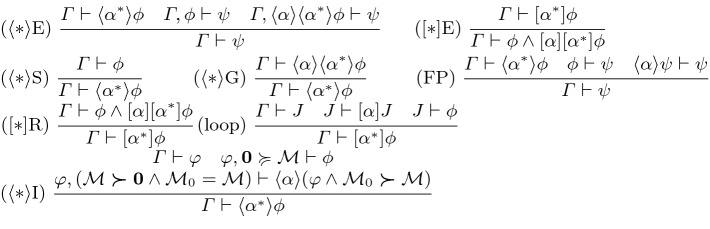
CdGL proof calculus: loops ($\mathcal {M}_0$ fresh)

Figure 4 provides rules for repetitions. In rule $\langle * \rangle$I, $\mathcal {M}$ indicates an arbitrary termination metric where $\varvec{\succ }$ and $\varvec{\succcurlyeq }$ denote strict and nonstrict comparison in an arbitrary (effectively) well-founded [28] partial order. Metavariable ${\varvec{0}}$ represents a terminal value at which iteration stops; we will choose ${\varvec{0}}= 0$ in our example, but ${\varvec{0}}$ need not be 0 in general. $\mathcal {M}_0$ is a fresh variable which remembers $\mathcal {M}$. Angel plays  by repeating an $\alpha$ strategy which always decreases the termination metric. Angel maintains a formula $\varphi$ throughout, and stops once $0 \varvec{\succcurlyeq }\mathcal {M}$. The postcondition need only follow from termination condition $0 \varvec{\succcurlyeq }\mathcal {M}$ and convergence formula $\varphi$. Simple real comparisons $x \ge y$ are not well-founded, but inflated comparisons like $x \ge y + 1$ are. Well-founded metrics ensure convergence in finitely (but often unboundedly) many iterations. In the simplest case, $\mathcal {M}$ is a real-valued term. Generalizing $\mathcal {M}$ to tuples enables, e.g., lexicographic termination metrics. For example, the metric in the proof of Example 4 is the distance to the goal, which must decrease by some minimum amount each iteration.

Repetition games can be folded and unfolded ($[*]$E, $[*]$R). Rule FP says  is a least pre-fixed-point. It works backwards: first show $\psi$ holds after  then preserve $\psi$ when each iteration is unwound. Rule loop is the repetition invariant rule. Demonic repetition is eliminated by $[*]$E.

Like any first-order program logic, CdGL proofs contain first-order reasoning at the leaves. Decidability of constructive real arithmetic is an open problem [33], so first-order facts are proven manually in practice. Our semantics embed CdGL into type theory; we defer first-order arithmetic proving to the host theory. Even effectively-well-founded $\varvec{\succcurlyeq }$ need not have decidable guards (${\varvec{0}}\varvec{\succ }\mathcal {M}\vee \mathcal {M}\varvec{\succcurlyeq }{\varvec{0}}$) since exact comparisons are not computable [6]. We may not be able to distinguish $\mathcal {M}= {\varvec{0}}$ from very small positive values of $\mathcal {M},$ leading to one unnecessary loop iteration, after which $\mathcal {M}$ is certainly ${\varvec{0}}$ and the loop terminates. Comparison up to $\varepsilon > 0$ is decidable [12] ($f > g \vee (f < g + \varepsilon )$).

**Fig. 5.**
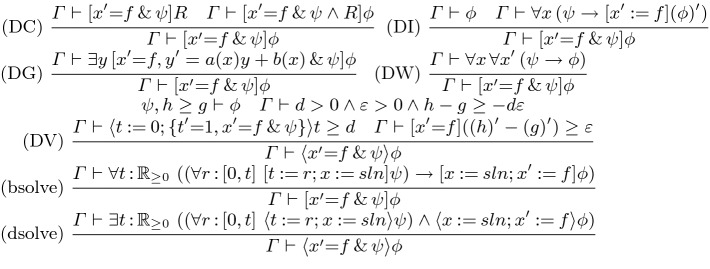
CdGL proof calculus: ODEs. In bsolve and dsolve, *sln* solves $x'=f$ globally, *t* and *r* fresh, $x' \notin \mathop {\text {FV}}(\phi )$

Figure 5 gives the ODE rules, which are a constructive version of those from  [42]. For nilpotent ODEs such as the plant of Example 4, reasoning via solutions is possible. Since CdGL supports nonlinear ODEs which often do not have closed-form solutions, we provide invariant-based rules, which are complete [46] for invariants of polynomial ODEs. *Differential induction* DI [41] says $\phi$ is an invariant of an ODE if it holds initially and if its *differential formula* [41] $(\phi )'$ holds throughout, for example $(f \ge g)' \equiv ((f)' \ge (g)')$. Soundness of DI requires differentiability, and $(\phi )'$ is not provable when $\phi$ mentions nondifferentiable terms. *Differential cut* DC proves *R* invariant, then adds it to the domain constraint. *Differential weakening* DW says that if $\phi$ follows from the domain constraint, it holds throughout the ODE. *Differential ghosts* DG permit us to augment an ODE system with a fresh dimension *y*,  which enables [46] proofs of otherwise unprovable properties. We restrict the right-hand side of *y* to be linear in *y* and (uniformly) continuous in *x* because soundness requires that ghosting ${y'}$ does not change the duration of an ODE. A linear right-hand side is guaranteed to be Lipschitz on the whole existence interval of equation ${x'} = f,$ thus ensuring an unchanged duration by (constructive) Picard-Lindelöf [34]. *Differential variants* [41, 53] DV is an Angelic counterpart to DI. The schema parameters *d* and $\varepsilon$ must not mention $x,{x'},t,{t'}$. To show that *f* eventually exceeds *g*,  first choose a duration *d* and a sufficiently high minimum rate $\varepsilon$ at which $h-g$ will change. Prove that $h-g$ decreases at rate at least $\varepsilon$ and that the ODE has a solution of duration *d* satisfying constraint $\psi$. Thus at time *d*,  both $h \ge g$ and its provable consequents hold. Rules bsolve and dsolve assume as a side condition that *sln* is the unique solution of ${x'}=f$ on domain $\psi$. They are convenient for ODEs with simple solutions, while invariant reasoning supports complicated ODEs.

## Theory: Soundness

Following constructive counterparts of classical soundness proofs for , we prove that the CdGL proof calculus is sound: provable formulas are true in the CIC semantics. For the sake of space, we give statements and some outlines here, reporting all proofs and lemmas elsewhere [10]. Similar lemmas have been used to prove soundness of  [45], but our new semantics lead to simpler statements for Lemmas 10 and 11. The coincidence property for terms is not proved but assumed, since we inherit a semantic treatment of terms from the host theory. Let  be *s* with the values of *x* and *y* swapped. Let  be ${{\textsf {set}}}\ {s}\ x\ (f\ s).$ Defined CIC term $s \overset{{V}}{=} t \leftrightarrow \texttt {*}_{x\in V}{(s\ x = t\ x)}$ says *s* and *t* agree on all $x \in V$.

### Lemma 9

**(Uniform renaming).** Let  rename *x* and *y* in proof term *M*.

- If  then .

### Lemma 10

**(Coincidence).** Assume $s\overset{{V}}{=} t$ where $V \supseteq \mathop {\text {FV}}(\varGamma ) \cup \mathop {\text {FV}}(\phi ).$

- If  then exists *N* such that .

### Lemma 11

**(Bound effect).** Let  and let $V\subseteq \mathop {\text {BV}}(\alpha )^\complement ,$ the complement of bound variables of $\alpha$.

- There exists *M* such that  iff there exists *N* such that .
- There exists *M* such that  iff there exists *N* such that .

### Definition 12

**(Term substitution admissibility** [40, **Def. 6**]**).** For a formula $\phi ,$ (likewise for context $\varGamma ,$ term *f*,  and game $\alpha$) we say  is *admissible* if *x* never appears free in $\phi$ under a binder of $\{x\} \cup \mathop {\text {FV}}(f)$.

### Lemma 13

**(Term substitution).** Let  substitute *f* for *x* in proof term *M*. Let  and  be admissible.

- If  then .

The converse implication also holds, though its witness is not necessarily *M*.

Soundness of the proof calculus follows from the lemmas, and soundness of the ODE rules employing several known results from constructive analysis.

### Theorem 14

**(Soundness).** If  holds, then sequent $(\varGamma \vdash \phi )$ is valid. As a special case, if  holds, then formula $\phi$ is valid.

### Proof Sketch

By induction on the derivation. The assignment case holds by Lemma 13 and Lemma 9. Lemma 10 and Lemma 11 are applied when maintaining truth of a formula across changing state. The equality and inequality cases of DI and DV employ the constructive mean-value theorem  [10, Thm. 21], which has been formalized, e.g., in Coq [17]. Rules DW, bsolve, and dsolve follow from the semantics of ODEs. Rule DC uses the fact that prefixes of solutions are solutions. Rule DG uses constructive Picard-Lindelöf [34], which constitutes an algorithm for arbitrarily approximating the solution of any Lipschitz ODE, with a convergence rate depending on its Lipschitz constant.    $\square$

We have shown that every provable formula is true in the type-theoretic semantics. Because the soundness proof is constructive, it amounts to an extraction algorithm from CdGL into type theory: for each CdGL proof, there exists a program in type theory which inhabits the corresponding type of the semantics.

## Theory: Extraction and Execution

Another perspective on constructivity is that provable properties must have witnesses. We show Existential and Disjunction properties providing witnesses for existentials and disjunctions. For modal formulas  and  we show proofs can be *used as* winning strategies: a big-step operational semantics $\textsf {play}$ allows playing strategies against each other to extract a proof that their goals hold in some final state *t*. Our presentation is more concise than defining the language, semantics, and properties of strategies, while providing key insights.

### Lemma 15

**(Existential Property).** Let . If  then there exist terms $f:\mathbb {R}$ and *N* such that .

### Lemma 16

**(Disjunction Property).** If  then there exists a proof term *N* such that  or .

The proofs follow their counterparts in type theory. The Disjunction Property considers truth at a *specific state*. Validity of $\phi \vee \psi$ does *not* imply validity of either $\phi$ or $\psi$. For example, $x < 1 \vee x > 0$ is valid, but its disjuncts are not.

Function $\textsf {play}$ below gives a big-step semantics: Angel and Demon strategies $\textsf {as}$ and $\textsf {ds}$ for respective goals $\phi$ and $\psi$ in game $\alpha$ suffice to construct a final state *t* satisfying both. By parametricity, *t* was found by playing $\alpha$, because $\textsf {play}$ cannot inspect *P* and *Q*,  thus can only prove them via $\textsf {as}$ and $\textsf {ds}$.Applications of $\textsf {play}$ are written $\textsf {play}_{\alpha }\ s\ \textsf {as}\ \textsf {ds}$ (*P* and *Q* implicit). Game consistency (Corollary 17) is by $\textsf {play}$ and consistency of type theory. Note that  is played by swapping the Angel and Demon strategies in $\alpha$. 
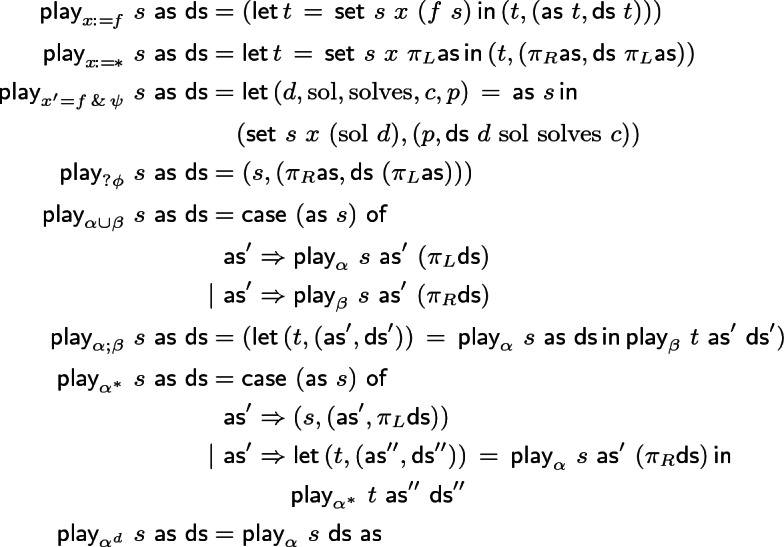


### Corollary 17

**(Consistency).** It is never the case that both  and  are inhabited.

### Proof

Suppose  and  then $\pi _{R}(\textsf {play}_{\alpha }\ s\ \textsf {as}\ \textsf {ds}) : \bot ,$ contradicting consistency of type theory.    $\square$

The $\textsf {play}$ semantics show how strategies can be executed. Consistency is a theorem which ought to hold in any GL and thus helps validate our semantics.

## Conclusion and Future Work

We extended Constructive Game Logic CGL to CdGL for constructive *hybrid* games. We contributed new semantics. We presented a natural deduction proof calculus for CdGL and used it to prove reach-avoid correctness of 1D driving with adversarial timing. We showed soundness and constructivity results.

The next step is to implement a proof checker, game interpreter, and synthesis tool for CdGL. Function $\textsf {play}$ is the high-level interpreter algorithm, while synthesis would commit to one Angel strategy and allow black-box Demon implementations for an external environment. Angel strategies are positive and are synthesized by extracting witnesses from each introduction rule. Demonic invariants and test conditions describe allowed observable behaviors. Demon strategies are negative and characterized by observable behaviors, so it suffices to monitor their compliance with invariants and test conditions extracted from the proof.
